# Cell Death Mechanisms in Tumoral and Non-Tumoral Human Cell Lines Triggered by Photodynamic Treatments: Apoptosis, Necrosis and Parthanatos

**DOI:** 10.1038/srep41340

**Published:** 2017-01-23

**Authors:** J. Soriano, I. Mora-Espí, M. E. Alea-Reyes, L. Pérez-García, L. Barrios, E. Ibáñez, C. Nogués

**Affiliations:** 1 Departament de Biologia Cel·lular, Fisiologia i Immunologia, Universitat Autònoma de Barcelona, Spain; 2 Departament de Farmacologia, toxicologia i Química Terapèutica and Institut de Nanociència i Nanotecnologia (IN2UB), Universitat de Barcelona, Spain

## Abstract

Cell death triggered by photodynamic therapy can occur through different mechanisms: apoptosis, necrosis or autophagy. However, recent studies have demonstrated the existence of other mechanisms with characteristics of both necrosis and apoptosis. These new cell death pathways, collectively termed regulated necrosis, include a variety of processes triggered by different stimuli. In this study, we evaluated the cell death mechanism induced by photodynamic treatments with two photosensitizers, *meso*-tetrakis (4-carboxyphenyl) porphyrin sodium salt (Na-H_2_TCPP) and its zinc derivative Na-ZnTCPP, in two human breast epithelial cell lines, a non-tumoral (MCF-10A) and a tumoral one (SKBR-3). Viability assays showed that photodynamic treatments with both photosensitizers induced a reduction in cell viability in a concentration-dependent manner and no dark toxicity was observed. The cell death mechanisms triggered were evaluated by several assays and cell line-dependent results were found. Most SKBR-3 cells died by either necrosis or apoptosis. By contrast, in MCF-10A cells, necrotic cells and another cell population with characteristics of both necrosis and apoptosis were predominant. In this latter population, cell death was PARP-dependent and translocation of AIF to the nucleus was observed in some cells. These characteristics are related with parthanatos, being the first evidence of this type of regulated necrosis in the field of photodynamic therapy.

Photodynamic therapy (PDT) is a therapeutic modality for the treatment of neoplastic and non-neoplastic diseases. It is based on the administration of a photosensitizer (PS) that accumulates in target tissues, followed by irradiation with visible light. The combination of PS, light and oxygen triggers photochemical processes leading to the formation of reactive oxygen species (ROS), which interact with cellular structures causing the selective destruction of the irradiated tissue^1^,^2^. Cell death triggered by PDT can occur through different mechanisms: apoptosis, necrosis, autophagy or mitotic catastrophe. The pathway that is activated after photodynamic treatments depends on the PS, treatment doses, subcellular localization of the PS and cell type^3^,^4^,^5^,^6^.

Traditionally, necrosis has been considered an unregulated process independent of apoptosis. However, recent studies have demonstrated novel mechanisms of cell death with characteristics of both apoptosis and necrosis, challenging this idea. The term “regulated necrosis” has been proposed by the Nomenclature Committee on Cell Death to comprise these mechanisms^7^,^8^, which occur in response to numerous damaging situations such as alkylating damage of DNA, exposition to certain excitotoxins or pathogens, the binding of some ligands to membrane receptors or ROS exposition^9^. However, it is important to take into account that these triggers are not exclusive of regulated necrosis because, depending on the cellular context, they can induce other cell death mechanisms such as apoptosis.

Van den Berghe *et al*. defined regulated necrosis as a genetically controlled cell death process that eventually results in cellular leakage, and that is morphologically characterized by cytoplasmic granulation, as well as organelle and/or cellular swelling (oncosis)^10^. Multiple subroutines of regulated necrosis, such as necroptosis, parthanatos, ferroptosis, autosis, netosis or pyroptosis, share these features, but they are triggered by different stimuli and their underlying molecular pathways are different^10^.

Parthanatos is one of the most studied mechanisms of regulated necrosis^11^,^12^. It is elicited by stimuli that induce DNA damage, such as ultraviolet irradiation, alkylating agents or ROS^13^, which overactivate Poly (ADP-ribose) polymerase (PARP), an (ADP-ribosyl) transferase involved in DNA repair. This hyperactivation of PARP induces a massive PARtylation of proteins, depleting cells of NAD^+^ and ATP and leading to an energetic catastrophe. In addition, PARP hyperactivation produces PAR polymers that induce the release of the truncated form of apoptosis-inducing factor (AIF) from the outer mitochondrial membrane and its entry into the nucleus, where AIF induces nuclear fragmentation through a still unknown mechanism.

In spite of the important role of ROS as an inductor of regulated necrosis, only a few studies have described regulated necrotic processes in response to photodynamic treatments^14^,^15^,^16^. The aim of this study was to evaluate the type of cell death mechanism induced by photodynamic treatment with two PSs, *meso*-tetrakis (4-carboxyphenyl) porphyrin sodium salt (Na-H_2_TCPP) and its derivative zinc (II) *meso*-tetrakis (4-carboxyphenyl) porphyrin sodium salt (Na-ZnTCPP) (Fig. 1). Experiments were performed in two human breast epithelial cell lines, a non-tumoral (MCF-10A) and a tumoral one (SKBR-3).

## Results

### Cytotoxicity of photodynamic treatments

The phototoxic effect of Na-H_2_TCPP and Na-ZnTCPP in MCF-10A and SKBR-3 cells is shown in Fig. 2. In absence of irradiation (dark toxicity, DT), the highest concentration of both PSs (4 μM) did not induce a significant decrease of cell viability at 24 h when compared with cells incubated without PSs. After 48 h, only MCF-10A cells treated with Na-H_2_TCPP showed a significant decrease of cell survival.

On the contrary, 10 min of irradiation with red light lead to a decrease in cell survival, in a porphyrin concentration dependent manner when compared with cells incubated without PSs and irradiated too, and some differences between porphyrins and cell lines were observed. At 24 h post-irradiation, MCF-10A cells treated with the lower concentrations (0.5 or 1 μM) of Na-H_2_TCPP or Na-ZnTCPP showed higher viability than SKBR-3 cells subjected to the same treatments. Treatments with the highest concentrations (2 and 4 μM) resulted in higher cytotoxicity and no significant differences between porphyrins or cell lines were observed. In SKBR-3 cell line the IC_50_ at 24 h was four times lower when treated with Na-H_2_TCPP and nearly thrice lower when treated with Na-ZnTCPP compared to MCF-10A line (Table 1).

At 48 h after photodynamic treatments, SKBR-3 cells treated with 0.5 μM Na-ZnTCPP showed a significantly lower viability than SKBR-3 cells treated with 0.5 μM Na-H_2_TCPP or MCF-10A cells treated with both PSs at the same concentration. Cell viability was also lower for SKBR-3 cells treated with 1 or 2 μM Na-H_2_TCPP than for MCF-10A cells subjected to the same treatments. Regarding treatments with the highest concentration (4 μM), no significant differences between porphyrins or cell lines were observed in cell survival, which was between 10–20% in all the cases. Hereafter, all experiments were performed using a working concentration of 4 μM Na-H_2_TCPP or Na-ZnTCPP, because this concentration showed the highest cytotoxicity in both cell lines.

### Nuclear morphology after photodynamic treatments

Three different nuclear morphologies of cells stained with H-33258 24 h after irradiation were identified (Fig. 3): necrotic, apoptotic and spotted nuclei. In MCF-10A cells treated with Na-H_2_TCPP, 28% of necrotic nuclei and 41% of spotted nuclei were observed. On the other hand, SKBR-3 cells incubated with Na-H_2_TCPP showed 40% of necrotic nuclei and 26% of nuclei with the classical apoptotic morphology.

Similar results were observed in cells treated with Na-ZnTCPP, but in this case the percentage of necrotic nuclei was significantly higher (50% in SKBR-3 and 54% in MCF-10A cells) than the percentage of apoptotic (17% in SKBR-3) or spotted nuclei (19% in MCF-10A).

### DNA fragmentation after photodynamic treatments

TUNEL assay was used to detect DNA fragmentation, characteristic of early stages of apoptotic processes (Fig. 4). MCF-10A cells treated with Na-H_2_TCPP or Na-ZnTCPP showed 41% and 24% of TUNEL-positive nuclei, respectively, 5 h after treatments. However, SKBR-3 cells treated with the same PSs showed lower percentages of TUNEL-positive nuclei (12 and 11%, respectively). Live cells and cells with a necrotic morphology did not exhibit TUNEL-positive staining.

### Phosphatidylserine translocation and plasma membrane integrity

At 2 h post-photodynamic treatments, cells were processed for Annexin V (ANV) and propidium iodide (PI) double staining (Fig. 5). ANV was used to detect the translocation of phosphatidylserine from the inner to the outer layer of the plasma membrane, an event that occurs during early apoptotic processes, whereas PI is an intercalating agent that only stains the nuclei of cells that have lost their plasma membrane integrity, as occurs in early necrotic process but not in early apoptosis. The majority of MCF-10A cells treated with either Na-H_2_TCPP or Na-ZnTCPP showed a double positive staining, which corresponds to necrotic cell death (66% and 55%, respectively). By contrast, most of SKBR-3 cells showed a double negative staining when treated with both PSs and only a small percentage of ANV+/PI− (8% in both cases) or ANV+/PI+ cells (5% in cells treated with Na-H_2_TCPP and 8% in cells treated with Na-ZnTCPP) was observed.

### Caspase-3 activation after photodynamic treatments

Activation of caspase-3 is a hallmark of apoptosis, serving as a convergence point for different apoptosis signaling pathways. In the present study it was evaluated 5 h after irradiation by immunofluorescence staining (Fig. 6). SKBR-3 cells treated with Na-H_2_TCPP or Na-ZnTCPP showed a positive staining of the cytoplasm in 26% and 16% of the cases, respectively. The majority of these positive cells also showed blebs on their plasma membrane and nuclear fragmentation, as expected for an apoptotic process. MCF-10A cells treated with Na-H_2_TCPP or Na-ZnTCPP showed 40% or 37% of stained cells, respectively, with spotted nuclei but no plasma membrane blebs.

### Effect of PARP-inhibition on the cytotoxicity of photodynamic treatments

The effect of different concentrations of 3-AB on cells irradiated in the presence of Na-H_2_TCPP or Na-ZnTCPP is shown in Fig. 7. In MCF-10A cells, the presence of 3-aminobenzamide (3-AB) during photodynamic treatments increased cell viability in a concentration dependent manner. The highest 3-AB concentration tested (2 mM) increased the survival of MCF-10A cells treated with Na-H_2_TCPP from 35% to 80% and from 30% to 54% in the case of MCF-10A cells incubated with Na-ZnTCPP. By contrast, incubation with 3-AB did not significantly alter the cytotoxic effect of the photodynamic treatments in SKBR-3 cells.

### Nuclear translocation of AIF

Translocation of AIF from mitochondria to the nucleus was assessed by immunofluorescence (Fig. 8). In control cells, AIF was apparently located inside the mitochondria. However, 5 h after irradiation MCF-10A cells treated with both PSs, showed a diffuse red fluorescence located in both the cytoplasm and the nucleus, which presented a spotted appearance. A similar result was observed in SKBR-3 cells. In both cell lines, cells with non-fragmented nuclei did not show AIF translocation.

## Discussion

In the present study we compared the photodynamic effect of two different PSs on non-tumoral and tumoral breast epithelial cell lines. A concentration dependent reduction of cell viability was induced in both cell lines by the treatment with Na-H_2_TCPP or Na-ZnTCPP and irradiation. However, the response to the treatment was different in both cell lines except for the highest concentration of the two PSs (4 μM), which triggered the death of most cells. At lower PSs concentrations, non-tumoral MCF-10A cells showed a higher resistance to photodynamic treatments than tumoral SKBR-3 cells. This result is of great interest for the development of selective photodynamic treatments, with less side effects in healthy tissues.

Notably, differences between cell lines in their response to photodynamic treatments, were also manifested as differences in nuclear morphologies. Specifically, SKBR-3 cells showed necrotic or apoptotic nuclei morphologies, whereas in MCF-10A cells the predominant morphologies were necrotic and spotted nuclei, which do not correspond with necrosis or apoptosis classical features. These results suggest that photodynamic treatments triggered different cell death pathways as a function of the cell line, as it has been described by other authors^3^,^4^,^17^.

In order to determine the cell death mechanisms activated by photodynamic treatments, TUNEL and ANV/PI assays and immunofluorescence staining of active caspase-3 were performed at short times after irradiation (5, 2 and 5 h, respectively).

Results showed significant differences between both cells lines when treated with Na-H_2_TCPP. On the one hand, SKBR-3 cells presented small numbers of TUNEL-positive nuclei, with a staining pattern similar to that observed in apoptotic nuclei, ANV+/PI+ and ANV+/PI− cells, and caspase-3 positive cells. These results suggest that photodynamic treatments with Na-H_2_TCPP induce a slow response in SKBR-3 cells that culminates in two different cell death mechanisms: necrosis and apoptosis. On the other hand, cell death events were triggered much faster in MCF-10A cells after photosensitization. At short times after irradiation, a high percentage of TUNEL-positive nuclei with a spotted staining pattern was found, as corresponds to an apoptotic process, but most of the cells were ANV+/PI+, characteristic of necrosis. In addition, a high number of caspase-3 positive cells was observed 5 h after irradiation, which is traditionally considered an event exclusively associated with apoptosis. A recent study has demonstrated that the treatment of human-derived neuroblastoma cells with chelerythrine triggered a regulated form of necrotic cell death induced by an increase of ROS levels, which elicited an overactivation of caspases, caspase-3 included^18^. Given that PDT induces the formation of ROS, it is possible that a similar caspase-3 activation occurred under our experimental conditions. All these features (fragmented and condensed nuclei, early loss of membrane integrity but no blebbing and TUNEL positive staining) are considered characteristics of regulated necrosis^9^.

Regulated necrosis includes a wide variety of cell death pathways that share characteristics of both apoptotic and necrotic processes. One of these pathways is parthanatos, which is characterized by nuclear fragmentation, early plasma membrane rupture, absence of membrane blebbing, mitochondrial depolarization, PARP-dependence and AIF translocation from the mitochondria to the nucleus (Table 2)^11^,^19^. In addition, caspase activation has been observed in this process, but it is not mandatory for the execution of parthanatos^11^.

As some of these features correspond with the characteristics of the MCF-10A cells with spotted nuclei observed after treatements with Na-H_2_TCPP, to confirm if parthanatos was involved in MCF-10A cell death, the PARP inhibitor 3-AB was used. Results showed that the presence of 3-AB increased the viability of MCF-10A cells treated with Na-H_2_TCPP, in a concentration dependent manner. Moreover, the higher concentration of 3-AB (2 mM) increase cell viability from 35% to 80%, which corresponds with the percentage of spotted nuclei, TUNEL positive and active caspase-3 positive cells observed in the previous experiments. A similar rescue-effect has been previously reported in glioma cells treated with deoxypodophyllotoxin in the presence of 3-AB^20^. Contrarily, in SKBR-3 cells the presence of 3-AB did not modify the photodynamic effect of Na-H_2_TCPP at any concentration, which supports the evidences of necrotic and apoptotic cell death in SKBR-3, because PARP is not involved in classic necrosis and its function is inhibited during apoptosis.

To further confirm that parthanatos was involved in cell death, AIF inmunostaining was performed 5 h after photodynamic treatments. In both cell lines treated with Na-H_2_TCPP, AIF translocation from the mitochondria to the nucleus was observed in cells showing nuclear fragmentation. This event is associated with apoptotic cell death^21^ and with parthanatos [11], but not with classic necrosis or other types of regulated necrosis.

Subcellular localization of the PSs could explain how parthanatos is triggered by photodynamic treatments, but under our experimental conditions it was not possible to visualize the PSs inside the cells. However, it has been described that H_2_TCPP colocalizes with late endosomes and it is also diffusely distributed all over the cytoplasm and nucleoplasm in colon adenocarcinoma WiDr cells^22^. Taking into account this evidence, we can hypotesize that photoactivation of Na-H_2_TCPP located in the nucleus of MCF-10A cells, could lead to a local increase of ROS, resulting in DNA damage and triggering PARP overactivation, the first step of parthanatos pathway. Nevertheless, SKBR-3 cells do not present the characteristics of parthanatos and a possible explanation for the different cell death mechanisms observed in both cells lines could be related to p53 expression. Recent studies have revealed that p53 is essential for PARP-mediated necrosis in response to DNA damage induced by ROS^23^,^24^, suggesting that p53 regulates PARP activation by transcriptional and post-translational events, and even by direct interaction^25^. MCF-10A cells express wild type p53, whereas SKBR-3 cells possess the structural mutant p53-R175H^26^,^27^. This mutation dramatically alters the conformation of p53, modifying its interactions with DNA and other proteins, which could explain why different cell death mechanisms are activated in the two cell lines. However, further studies should be performed in order to elucidate the possible role of p53 in our photodynamic treatments.

Finally, regarding to treatments with Na-ZnTCPP we observed similar results to those obtained with Na-H_2_TCPP, but the classical necrotic features prevailed over apoptotic (SKBR-3 cells) or regulated necrotic characteristics (MCF-10A cells).

Understanding the cell death mechanism/s induced by photodynamic treatments is important to develop more efficient and personalized treatments avoiding tumoral resistances. In this sense, our study demonstrates that the same photodynamic treatment can induce different cell death responses depending on the cell line studied. Moreover, the inhibition of PARP rescue most of the non-tumoral cells from cell death but has no significant effect on tumoral cells, which could be of great interest for the development of treatments with less side effects in healthy tissues. Finally, as far as we know, this study shows the first evidence of parthanatos induced by photosentization.

## Material and Methods

### Porphyrins Na-H_2_TCPP and Na-ZnTCPP

**Figure 1:**
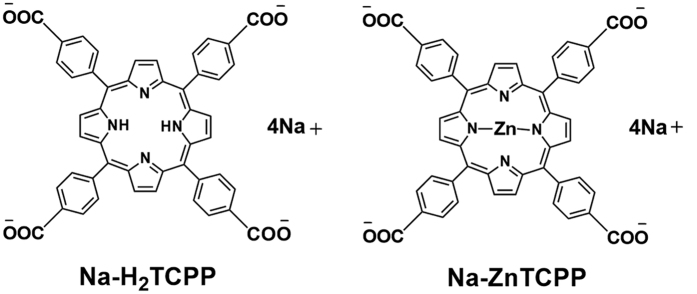
Structure of the porphyrins Na-H_2_TCPP and Na-ZnTCPP.

**Figure 2:**
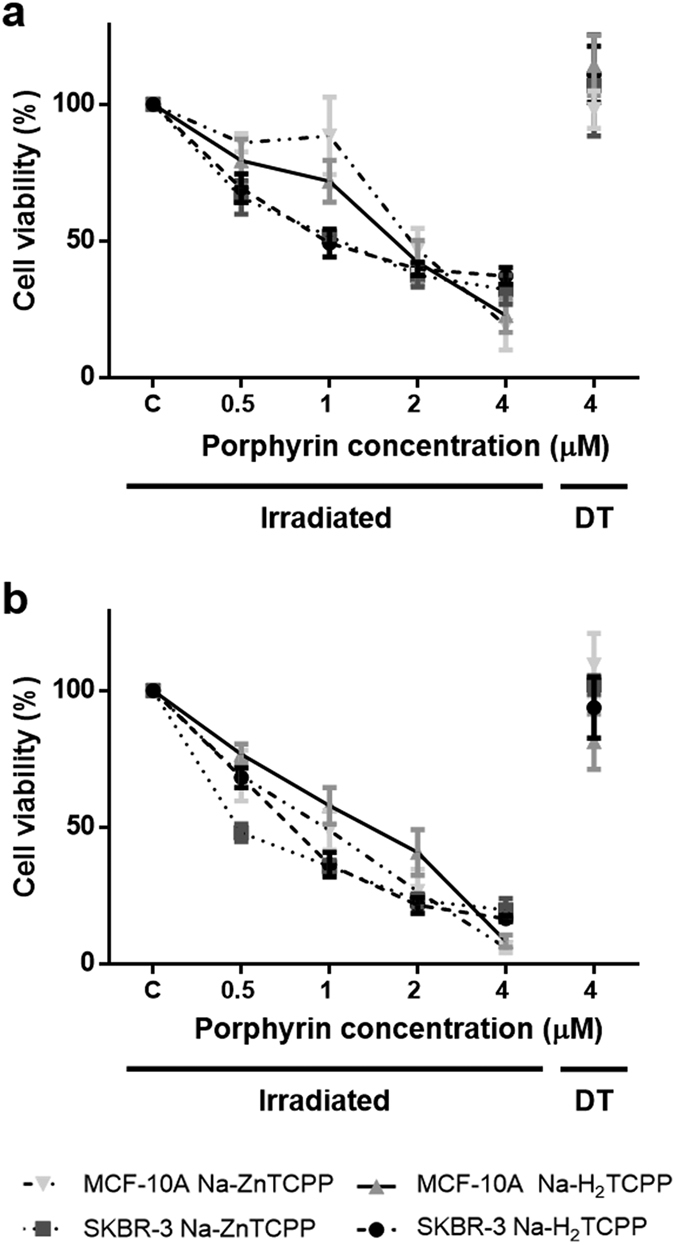
Viability of MCF-10A and SKBR-3 cells measured by MTT assay 24 **(a)** or 48 h **(b)** after photodynamic treatments. Cells were incubated with different concentrations of Na-H_2_TCPP or Na-ZnTCPP and irradiated 10 min with red light or kept in absence of light in order to evaluate the dark toxicity (DT) of the photosensitizers.

**Figure 3:**
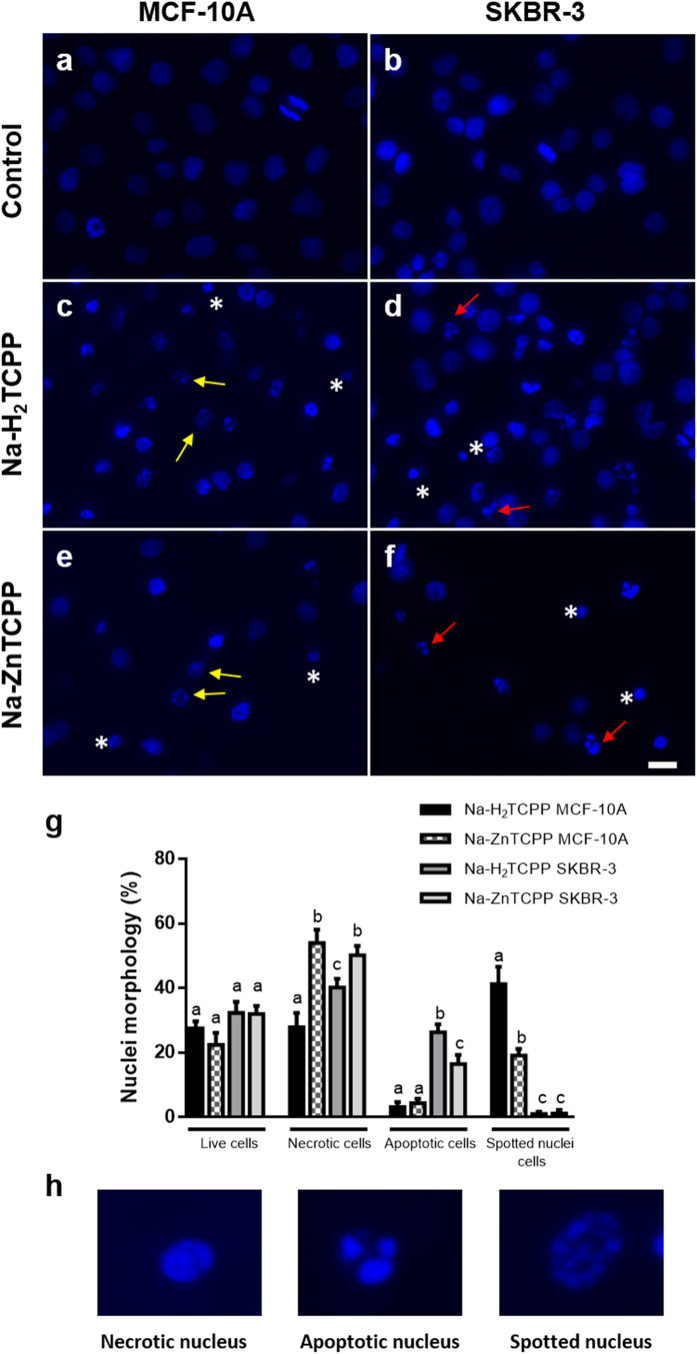
Nuclear morphology of Hoescht-33258 stained MCF-10A or SKBR-3 cells 24 h after photodynamic treatments. (**a,b)** Irradiated cells without photosensitizers (Control). (**c,d)** Cells treated with 4 μM Na-H_2_TCPP. (**e,f)** Cells incubated with 4 μM Na-ZnTCPP. Asterisks, red arrows and yellow arrows point to some representative necrotic, apoptotic and spotted nuclei, respectively. Scale bar, 20 μm. (**g)** Percentage of live, necrotic, apototic and spotted nuclei cells observed 24 h after photodynamic treatments with the two photosensitizers at a 4 μM concentration. Different superscripts on top of the columns denote significant differences between groups not sharing the same superscript within the same nuclear morphology. (**h)** Detail of the three nuclear morphologies of cell death observed.

**Figure 4:**
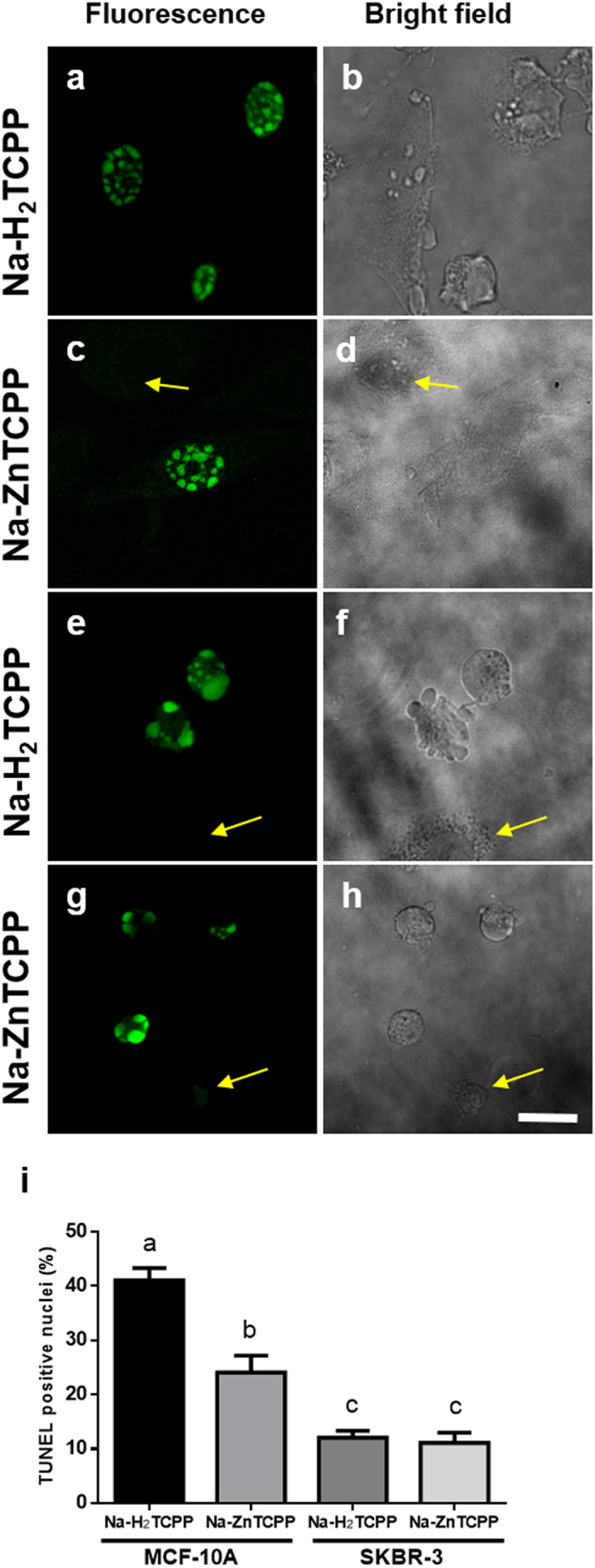
Cells processed for TUNEL assay 5 h after irradiation and observed under fluorescence and bright field microscope. (**a,b)** MCF-10A cells incubated with 4 μM Na-H_2_TCPP. (**c,d)** MCF-10A cells treated with 4 μM Na-ZnTCPP. (**e,f)** SKBR-3 cells incubated with 4 μM Na-H_2_TCPP. (**g,h)** SKBR-3 cells treated with 4 μM Na-ZnTCPP. Yellow arrows indicate TUNEL-negative cells. Scale bar, 20 μm. (**i)** Percentage of TUNEL-positive cells observed 5 h after photodynamic treatments with 4 μM Na-H_2_TCPP or Na-ZnTCPP. Different superscripts on top of the columns denote significant differences between groups not sharing the same superscript.

**Figure 5:**
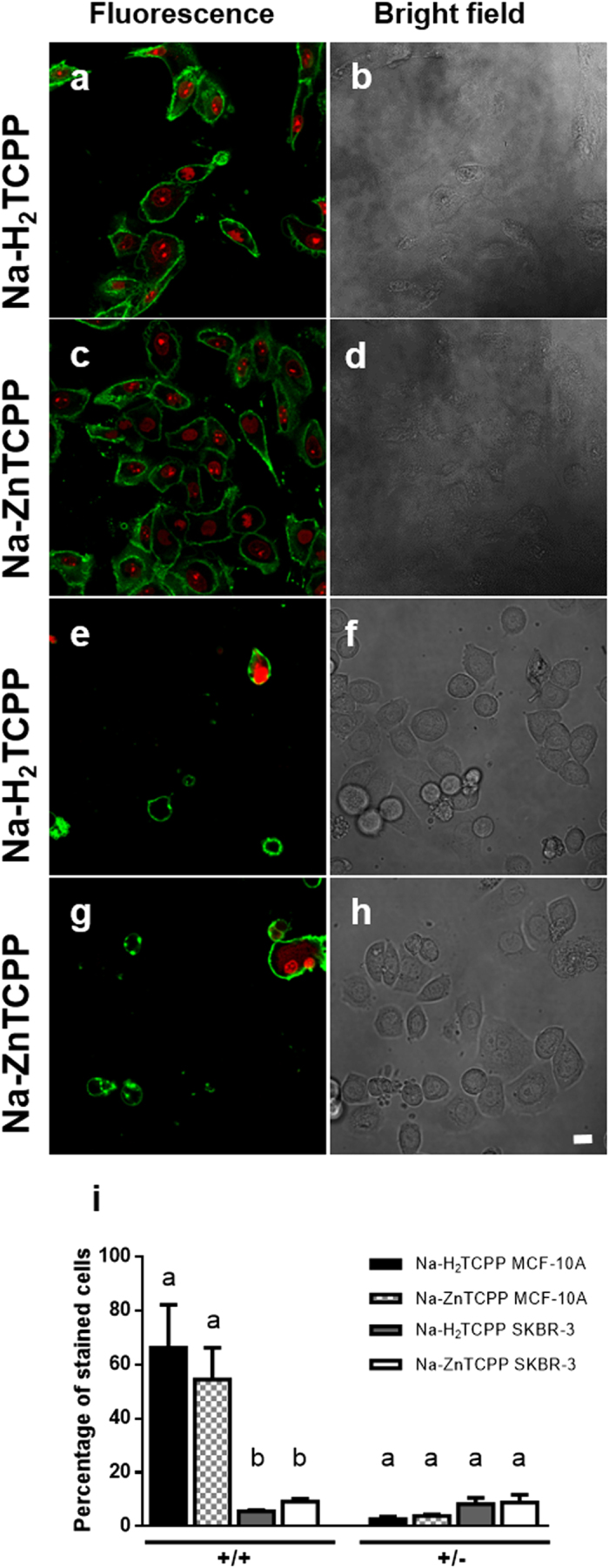
Cells processed with Annexin-V-FLUOS Staining Kit 2 h after irradiation and observed under fluorescence or bright field microscope. (**a,b)** MCF-10A cells incubated with 4 μM Na-H_2_TCPP. (**c,d)** MCF-10A cells treated with 4 μM Na-ZnTCPP. (**e,f)** SKBR-3 cells incubated with 4 μM Na-H_2_TCPP. (**g,h)** SKBR-3 cells treated with 4 μM Na-ZnTCPP. Green fluorescence corresponds to Annexin-V-FLUOS (ANV) and red fluorescence to propidium iodide (PI). Scale bar, 20 μM. (**i)** Percentage of ANV+/PI+ or ANV+/PI− cells observed 2 h after photodynamic treatments with 4 μM Na-H_2_TCPP or Na-ZnTCPP. Different superscripts on top of the columns denote significant differences between groups not sharing the same superscript within the cells +/+ and +/− for ANV/IP assay.

**Figure 6:**
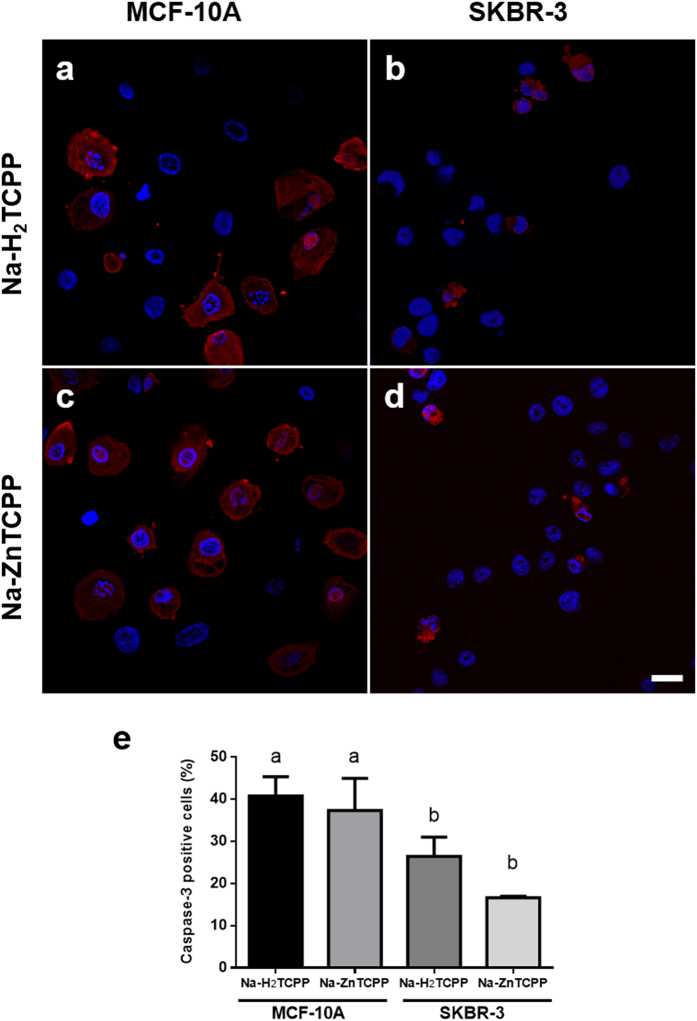
Cells processed for immunofluorescence staining of active caspase-3 (red) and counterstained with Hoescht-33258 (blue). (**a**) MCF-10A cells incubated with 4 μM Na-H_2_TCPP and processed 5 h after irradiation. (**b)** SKBR-3 cells treated with 4 μM Na-H_2_TCPP and processed 5 h after treatment. (**c)** MCF-10A cells incubated with 4 μM Na-ZnTCPP and processed 5 h after photodynamic treatment. (**d)** SKBR-3 cells treated with 4 μM Na-ZnTCPP and processed 5 h after treatment. Scale bar, 20 μm. (**e)** Percentage of active caspase-3 positive cells observed 5 h after photodynamic treatments. Different superscripts on top of the columns denote significant differences between groups not sharing the same superscript.

**Figure 7:**
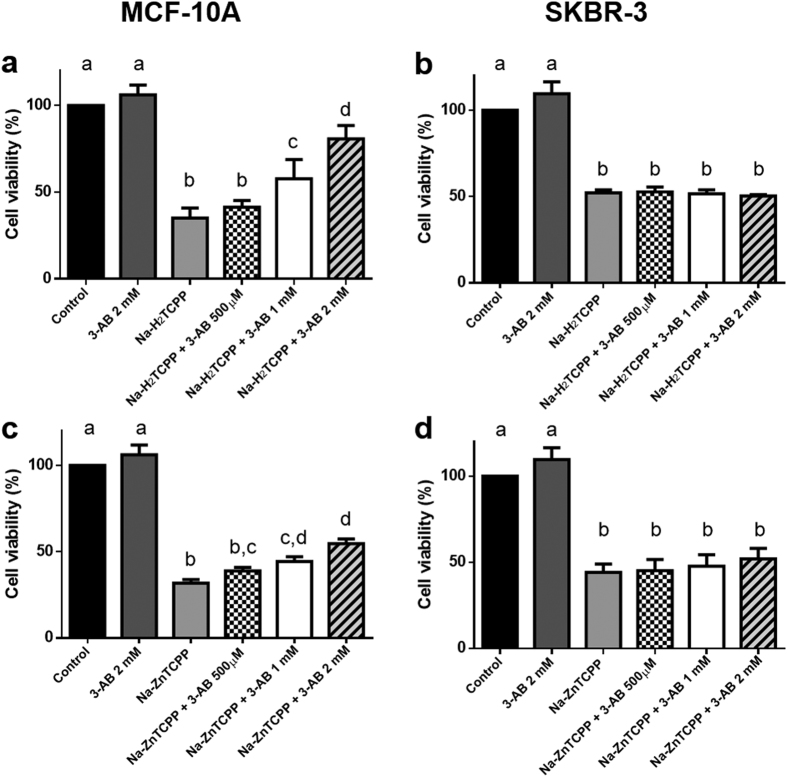
Viability of MCF-10A **(a,c)** and SKBR-3 cells **(b,d)** measured by MTT assay 24 h after photodynamic treatments in the presence of different concentrations of 3-Aminobenzamide and/or 4 μM Na-H_2_TCPP **(a,b)** or 4 μM Na-ZnTCPP **(c,d).** Different superscripts on top of the columns denote significant differences between groups not sharing the same superscript.

**Figure 8:**
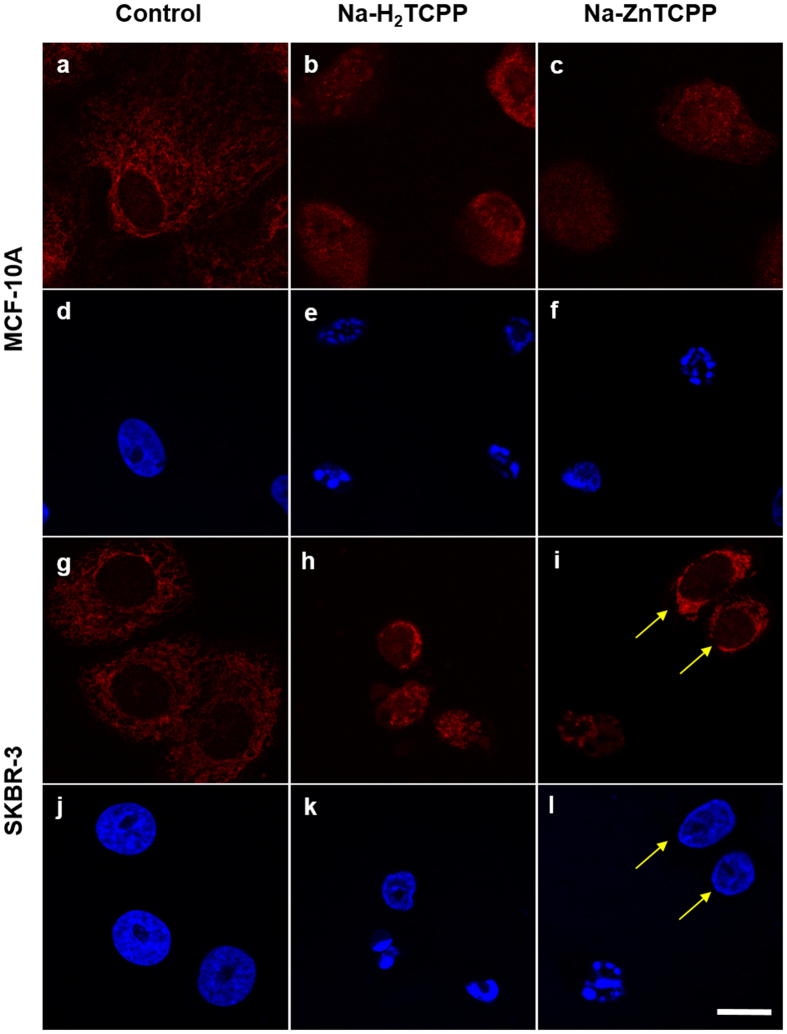
Cells incubated with Na-H_2_TCPP or Na-ZnTCPP for 3 h, irradiated 10 min and processed 5 h after photodynamic treatments for immunofluorescence staining of AIF (red) and counterstained with Hoescht-33258 (blue). (**a,d)** MCF-10A control cells. (**b,e)** MCF-10A cells treated with 4 μM Na-H_2_TCPP. (**c,f)** MCF-10A cells treated with 4 μM Na-ZnTCPP. (**g,j)** SKBR-3 control cells. (**h,k)** SKBR-3 cells treated with 4 μM Na-H_2_TCPP. (**i,l)** SKBR-3 cells treated with 4 μM Na-ZnTCPP. Yellow arrows correspond to cells with unaltered nuclei. Scale bar, 20 μm.

The structure of the Porphyrins Na-H_2_TCPP and Na-ZnTCPP is shown in Fig. 1, and their synthesis has been reported elsewhere^28^,^29^.

### Cell culture

Experiments were conducted with two different human mammary epithelial cell lines, a non-tumorigenic (MCF-10A) and a tumorigenic one (SKBR-3). Both cell lines were purchased from American Type Culture Collection (ATCC, Manassas, VA, USA). The MCF-10A cell line was cultured in DMEM/F12 (Gibco, Paisley, United Kingdom) supplemented with 5% horse serum (Gibco), 20 ng/ml epidermal growth factor (Gibco), 0.5 mg/ml hydrocortisone (Sigma-Aldrich), 100 ng/ml cholera toxin (Sigma-Aldrich) and 10 μg/ml insulin (Gibco). The SKBR-3 adenocarcinoma cell line was cultured in McCoy’s 5A modified medium (Gibco) supplemented with 10% fetal bovine serum (Gibco). Both cell lines were maintained at 37 °C and 5% CO_2_ (standard conditions).

For each experiment, cells were seeded in 24-well dishes, with or without coverslips, at a density of 50,000 cells/well. Treatments were performed 24 h after seeding.

### Photodynamic treatments

Cells were incubated in serum-free medium with different concentrations of Na-H_2_TCPP or Na-ZnTCPP (0.5, 1, 2 and 4 μM) for 3 h. Next, cells were washed thrice with Phosphate-Buffered Saline (PBS) and maintained in culture medium during irradiation and post-treatment. Irradiation was performed for 10 min using a PhotoActivation Universal Light device (PAUL, GenIUL, Barcelona, Spain), in the range of 620–630 nm (red light) and with a mean intensity of 55 mW/cm^2^.

### MTT assay

Cell viability was determined 24 or 48 h after treatments by the 3-(4,5-dimethylthiazol-2-yl)-2,5diphenyltetrazolium bromide (MTT) assay (Sigma-Aldrich). The absorbance was recorded at 540 nm using a Victor 3 Multilabel Plate Reader (PerkinElmer, Waltham, MA, USA). To evaluate the toxicity of the PSs in absence of irradiation (Dark toxicity, DT), cells were also incubated with the maximum concentration of Na-H_2_TCPP or Na-ZnTCPP (4 μM) and were kept in dark conditions. For each treatment, viability was calculated as the absorbance of treated cells in comparison with control cells. Three independent experiments were performed in each case.

### Hoechst Staining and nuclear morphology analysis

Nuclear staining was performed 24 h after treatments. Cells grown on coverslips were fixed in cold methanol for 5 min, air dried and stained with 5 μg/ml Hoescht-33258 (H-33258, Life Technologies, Carlsbad, CA) for 3 min. Preparations were mounted in ProLong Gold (Life Technologies) and observed by fluorescence microscopy (Olympus IX70, Olympus, Hamburg, Germany). Previously described morphological criteria^14^,^30^ were used to assess classic necrotic (small round shaped nuclei with highly condensed chromatin), regulated necrotic (nuclei with highly condensed chromatin spots, variable in number and shape) and apoptotic cells (nuclear shrinkage and fragmentation with apoptotic body formation). Hereafter, we will refer to the nuclear morphology of regulated necrotic cells as spotted nuclei.

### TUNEL Assay

To detect DNA fragmentation, TUNEL assay was performed 5 h after the treatments. Cells grown on coverslips were fixed in 4% paraformaldehyde/PBS (Sigma-Aldrich) for 15 min at 4 °C, washed thrice with PBS, permeabilized with 0.1% Triton X-100 (Sigma-Aldrich) in PBS for 2 min and incubated with TUNEL reaction mixture (Roche, Indianapolis, IN, USA) for 1 h at 37 °C. Cells were washed with PBS, mounted in ProLong Gold and observed under a Confocal Laser Scanning Microscope (CLSM, Olympus XT7).

### Annexin V/Propidium Iodide Assay

Phosphatidylserine translocation and membrane integrity was evaluated using Annexin-V-FLUOS staining kit (Roche) 2 h after the photodynamic treatments. The protocol was performed in accordance with manufacturer recommended conditions and samples were analyzed under a CLSM.

### 3-Aminobenzamide Treatments

Cells were incubated for 3 h in serum-free medium with different concentrations (0.5, 1 and 2 mM) of a specific poly (ADP-ribose) polymerase (PARP) inhibitor, 3-aminobenzamide (3-AB, Sigma-Aldrich), in the presence or absence of 4 μM Na-H_2_TCPP or Na-ZnTCPP. The samples were then washed thrice with PBS, irradiated for 10 min in the presence of 3-AB and incubated in the same medium without serum overnight. MTT assays were performed 24 h after irradiation.

### Immunostaining

At 5 h of irradiation, cells grown on coverslips were fixed in 4% paraformaldehyde/PBS for 5 min, washed with PBS three times, permeabilized with 0.1% Triton X-100 in PBS and blocked with 5% BSA (Sigma-Aldrich) in PBS for 30 min. Cells were incubated for 1 h at 37 °C with either rabbit anti-apoptosis-inducing factor (AIF) polyclonal antibody (1:1000; Abcam, Cambridge, MA, USA. Ref: ab1998) or rabbit anti-active caspase 3 polyclonal antibody (1:1000; Sigma-Aldrich. Ref: C8487). Then, cells were washed with PBS three times and incubated 1 h at 37 °C with Alexa 594-conjugated goat anti-rabbit IgG antibody (1:2000; Life Technologies. Ref: A-11012). Finally, cells were washed thrice with PBS, counterstained with 5 μg/ml H-33258 for 3 min, mounted in Prolong Gold and analyzed under a CLSM.

### Statistical analyses

In order to quantify cell death morphologies, TUNEL positive nuclei, ANV/IP positive cells and active caspase-3 stained cells, three different and independent experiments were performed in each case and at least 250 cells were counted.

All graphics and statistical analyses were performed using GraphPad Prism version 6.00 for Windows, (GraphPad Software, La Jolla, California, USA). Analysis of variance (ANOVA) was run to test the effects of Na-H_2_TCPP or Na-ZnTCPP on different cellular parameters. On the one hand, the results of TUNEL assay and active caspase-3 immunofluorescence assay were evaluated through one-way ANOVA. On the other hand, the effects of treatments with both PSs on cell viability, nuclear morphology or ANV/IP assay were determined through two-way ANOVA. In all cases, the minimal significance level was set at P ≤ 0.05. Data are shown as mean ± standard error of the mean.

Significance is represented in the figures using an alphabetical superscript system on top of the columns. Letters shared in common between or among the groups would indicate no significant differences whereas different letters indicate statistically significant differences between groups.

## Additional Information

**How to cite this article**: Soriano, J. *et al*. Cell Death Mechanisms in Tumoral and Non-Tumoral Human Cell Lines Triggered by Photodynamic Treatments: Apoptosis, Necrosis and Parthanatos. *Sci. Rep.* **7**, 41340; doi: 10.1038/srep41340 (2017).

**Publisher's note:** Springer Nature remains neutral with regard to jurisdictional claims in published maps and institutional affiliations.

## Figures and Tables

**Table 1:** IC_50_ values of SKBR-3 and MCF-10A treated with Na-H_2_TCPP and Na-ZnTCPP, at 24 h after irradiation.

	MCF-10A	SKBR-3
Na-H_2_TCPP	2.255	0.518
Na-ZnTCPP	1.813	0.585

**Table 2:** Main features of apoptosis, necrosis and parthanatos.

	Apoptosis	Necrosis	Parthanatos
Plasma Membrane	Blebbing	Swelling and lysis	Lysis but no blebbing
ANV/PI assay	+/−	+/+	+/+
TUNEL assay	+	−	+
Caspase-3 activation	+	−	+ (not mandatory)
PARP dependence	−	−	+
AIF	Translocation to the nucleus (not always)	No translocation	Translocation to the nucleus
